# Discovery of Selective Small-Molecule Activators of a Bacterial Glycoside Hydrolase**

**DOI:** 10.1002/anie.201407081

**Published:** 2014-10-07

**Authors:** John F Darby, Jens Landström, Christian Roth, Yuan He, Gideon J Davies, Roderick E Hubbard

**Affiliations:** York Structural Biology Laboratory, Department of Chemistry, University of YorkYork YO10 5DD (UK)

**Keywords:** enzyme catalysis, glycoside hydrolase, glycosylation, inhibitors, protein structures

## Abstract

Fragment-based approaches are used routinely to discover enzyme inhibitors as cellular tools and potential therapeutic agents. There have been few reports, however, of the discovery of small-molecule enzyme activators. Herein, we describe the discovery and characterization of small-molecule activators of a glycoside hydrolase (a bacterial O-GlcNAc hydrolase). A ligand-observed NMR screen of a library of commercially available fragments identified an enzyme activator which yielded an approximate 90 % increase in *k*_cat_/*K*_M_ values (*k*_cat_=catalytic rate constant; *K*_M_=Michaelis constant). This compound binds to the enzyme in close proximity to the catalytic center. Evolution of the initial hits led to improved compounds that behave as nonessential activators effecting both *K*_M_ and *V*_max_ values (*V*_max_=maximum rate of reaction). The compounds appear to stabilize an active “closed” form of the enzyme. Such activators could offer an orthogonal alternative to enzyme inhibitors for perturbation of enzyme activity in vivo, and could also be used for glycoside hydrolase activation in many industrial processes.

Small-molecule enzyme inhibitors account for approximately 50 % of approved drugs[1] and can also be powerful chemical tools for probing biological systems. The majority of these inhibitors displace either substrate or cofactor, providing a clear target for ligand discovery. There are relatively few examples, however, of small-molecular activators of enzyme activity. Recent examples illustrate that a variety of mechanisms lead to activation, such as binding to known allosteric sites as for adenosine 5′-monophosphate (AMP)-activated protein kinase (AMPK)[2] and glucokinase,[3] interfering with binding close to the substrate site as for aldehyde dehydrogenase 2 (ALDH2),[4] and often with an unknown mechanism, such as for sirtuin-1 (SIRT1).[5] These diverse mechanisms of activation can be unpredictable and, therefore, pose a challenge for directed ligand discovery.

Recently, fragment-based lead discovery (FBLD) has been shown to be an effective method of generating probes, inhibitors, and drug candidates for a wide variety of protein targets.[6] FBLD begins by identifying low molecular weight compounds that interact with the target protein from a library of small molecules. This is done using biophysical techniques that are sensitive to weak molecular interactions with dissociation constant (*K_d_*) values in the millimolar range.[7] These initial fragment hits are then evolved to larger, higher affinity lead compounds.

The post-translational covalent attachment of *N*-acetylglucosamine (GlcNAc) to the hydroxy groups of serine and threonine is essential for life in metazoans. This *O*-GlcNAc modification is regulated by two enzymes: *O*-GlcNAc transferase (OGT) appends GlcNAc to serine and threonine whilst the *O*-GlcNAc hydrolase (OGA), removes GlcNAc from modified proteins.[8] The enzyme OGA has been the focus of much inhibitory work especially in the context of Alzheimer′s disease.[9] Most enzyme inhibition strategies for the OGA enzyme have been based upon rational mimicry of the reaction transition state and/or other stable species along the reaction coordinate,[10] designed from structural studies of a series of bacterial OGA homologues, such as enzymes from *Bacteroides thetaiotaomicron*[11] and *Clostridium perfringens*,[12] with few reports on library screening (see Ref. [13] for a notable example).

Our study was initially motivated by the prospect of screening fragment libraries for inhibitors of the *O*-GlcNAc hydrolase from *B. thetaiotaomicron*, termed BtGH84. However, the screen led to the discovery, analysis, and evolution of small-molecule enzyme activators. The compounds bind close to the active site, appear to stabilize an active “loop closed” conformation whilst also destabilizing the *apo* (unbound) structure. Kinetic analysis shows that the initial compound yields a twofold improvement in *k*_cat_/*K*_M_ values (*k*_cat_=catalytic rate constant, *K*_M_=Michaelis constant), whilst further activators identified through an analogue-by-catalogue approach result in up to eightfold improvements in *k*_cat_/*K*_M_ values. These novel compounds are the first activators for any glycoside hydrolase and an unexplored concept in carbohydrate chemistry and glycobiology.

We have previously described the generation of a fragment library containing small compounds with molecular weight (MW) less than 250 Da, selected for their facility to be improved by analogue-by-catalogue exploration of hit compounds.[14] The current 100 compound library was screened for binding to BtGH84 using three ligand-observed NMR spectroscopy experiments: saturation transfer difference (STD) NMR,[15] PO-WaterLOGSY,[16] and T1ρ-filter.[17] Fragments that showed evidence of binding in all three experiments were considered true hits for subsequent characterization. NMR spectroscopy experiments were repeated following addition of the known *O*-GlcNAcase inhibitor PUGNAc (**1**), which allows categorization of the hit compounds as either competitive or noncompetitive, that is, binding nonspecifically or to alternate sites.

In the initial screen, 22 fragments showed binding in all three experiments, of which 18 fragments were competitive with PUGNAc (See Figure S1 in the Supporting Information). Surprisingly, a comparison of the intensities of the NMR resonance signals indicates that the 4 noncompetitive hits bind more strongly to BtGH84 in the presence of PUGNAc, suggesting a cooperative effect on ligand binding (Figure S2). Kinetic characterization of hit compounds, using both 4-methylumbelliferyl-*N*-acetyl glucosamine (4MU-GlcNAc) and subsequently *p*-nitrophenyl-*N*-acetylglucosamine (*p*NP-GlcNAc) as a substrate, yielded measurable IC_50_ values for four compounds (Figure S1). Interestingly, 4-ethoxyquinazoline (**2**) showed an apparent activation of BtGH84 with an AC_50_ value of 3.5±1 mm (Figure 1; AC_50_=concentration of compound eliciting 50% of maximal activation). To our knowledge, this is the first experimentally discovered small molecule activator of a glycoside hydrolase which has been reported.

**Figure 1:**
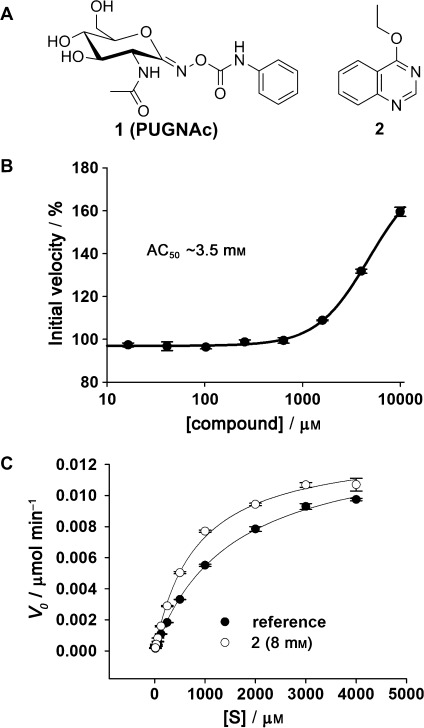
A) Chemical structures of the inhibitor PUGNAc (1), and the activator 2 identified by fragment screening. B) 4MU-GlcNAc cleavage assay AC_50_ curve for activator 2. Substrate concentration=500 μm. C) 4MU-GlcNAc cleavage assay Michaelis–Menten plot for BtGH84 in the absence and presence of 2. [S]=substrate concentration.

The affinity of **2** to BtGH84 was measured by NMR spectroscopy using proton relaxation rates R_2_s.[18] Activator **2** was titrated into a constant concentration of BtGH84, in the presence of PUGNAc, and proton R_2_ rates of **2** were measured (Figure S3) giving a *K*_d_ value of 3.1±0.7 mm. In the absence of PUGNAc the affinity of **2** for BtGH84 was weaker and could not be accurately estimated by this method. Michaelis–Menten kinetics of BtGH84 in the presence of **2** (at 8 mm) gave a decrease in the apparent *K*_M_ value (*K*_M(app)_) from 1.7±0.1 mm to 0.87±0.07 mm with a resultant increase in *k*_cat(app)_/*K*_M(app)_ values from 28 000±1800 to 54 000±1700 m^−1^ s^−1^ (Figure 1). Furthermore, as implied from the initial NMR data, binding of **2** seemed to increase the affinity of BtGH84 for PUGNAc (**1**). Isothermal titration calorimetry (ITC) in the absence and presence of **2** showed a decrease in the *K*_d_ value for PUGNAc from 3.9±0.12 μm to 1.1±0.24 μm (Figure S4) whilst kinetic analysis also revealed a similar decrease in the IC_50_ value for PUGNAc in the presence of **2** (Figure S4).

The single-crystal X-ray structure of BtGH84 was determined at a resolution of 2.2 Å (Table S1) by soaking apo-BtGH84 crystals with a mixture of **2** (50 mm) and PUGNAc (**1**; 10 mm). Electron density was clear for both the inhibitor PUGNAc and **2** bound in adjacent positions (Figure 2). Activator **2** appears to bind close to the active site, forming a π-stacking interaction on top of the Tyr137 residue (Y137) and a hydrogen bond to the Arg347 residue (R347).

**Figure 2:**
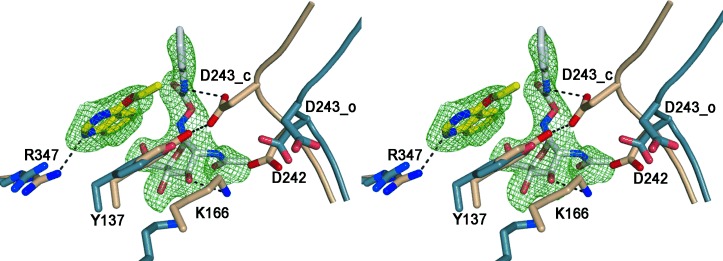
Stereo image of the activator and PUGNAc binding sites on BtGH84. The protein backbone of the catalytic loop is shown schematically with key active site residues in stick representation. The activator 2-bound structure (4UR9) is shown in fawn and the “loop open” structure (formed in the presence of streptozotocin which does not induce the closed conformation) is shown in blue (2W4X), overlaid on common secondary structure. The ligands are shown in stick representation, with carbon atoms of PUGNAc (1) in white and of activator 2 in yellow with the SA-Fo-Fc omit map contoured at 3σ r.m.s. (root mean square). The activator stacks on top of Y137 and forms one H-bond to R347 (H-bonds shown as dashed lines) thereby stabilizing the hydrogen bond between Y137 and D243. In the open structure (blue) the loop containing the catalytic residues D242 and D243 is shifted away from the active site and the H-bond between Y137 and D243 is lost.

An “analogue-by-catalogue” approach was used to identify similar compounds to **2** which were assessed in the enzyme assay. Replacement of the ethoxy group of **2** by a hydroxy function yielded an inactive analogue. Other available compounds that retained the ethoxy group but which contained conservative changes to the quinazoline ring, such as methyl or halogen substituents, were also inactive or less active than the initial molecule. Attempts to alter the core ring were more successful and led to a group of thienopyrimidine activators of BtGH84. Compound **3** retains the apparently important ethoxypyrimidine core (Table 1) and generated a better activator of BtGH84 in comparison to **2** with a lower AC_50_ value of 575±78 μm, a similar *K*_M(app)_ value, and improved *k*_cat(app)_/*K*_M(app)_ of 92 000±9600 m^−1^ s^−1^ (Table 1).

**Table 1:** Activator data from a 4MU-GlcNAc cleavage assay for activators 3–6 containing a thienopyrimidine core. 
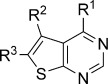

Compound	R^1^	R^2^	R^3^	AC_50_ [μm][a]	Maximum activation [%][a]	*K*_M(app)_ [μm][b]	*V*_max(app)_ [μmol min^−1^][b]	*k*_cat(app)_/*K*_M(app)_ [m^−1^ s^−1^][b]
–	–	–	–	–		1700±110	0.014±0.001	29 000±1800
**3**	-OEt	-Me	-Me	575±78	284	809±120	0.022±0.001	92 000±9600
**4**	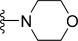	-Me	-Me	259±24	371	416±48	0.026±0.002	210 000±24 000
**5**	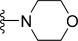	-H	-Et	274±12	329	643±47	0.024±0.001	125 000±9200
**6**	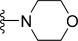	-H	-H	702±85	234	649±64	0.015±0.002	77 500±7700

[a] AC_50_ values and maximum activation obtained with a titration of activator molecule from 50 μm to 2 mm(substrate concentration=500 μm).

[b] Apparent kinetic values obtained using a Michaelis–Menten fit with 1 % DMSO across a substrate titration from 50 μm to 2 mm (activator concentration=2 mm).

Efforts to obtain a single-crystal X-ray structure of **3** with or without PUGNAc were not successful, perhaps reflecting the poor aqueous solubility of **3** compared to the original activator **2**. Further exploration of these compounds led to **4**, **5**, and **6** with a morpholino group at the R^1^ position. These activators had comparable values of maximum activation to **3** with slightly lower AC_50_ values (Table 1). However, **4** and **5** demonstrated significantly improved *k*_cat(app)_/*K*_M(app)_ values approaching eight times the rate constant of the unactivated enzyme (Table 1). In the absence of crystal structures, binding of the thienopyrimidine activator **4** was assessed by STD NMR (Figure S6). Saturation transfer from BtGH84 to **4** was significantly diminished in the presence of **2** (at 25 mm), suggesting that the two activators indeed compete for the same binding site.

The influence of fragment binding on protein stability was assessed by differential scanning fluorimetry (DSF), measuring the melting temperature (*T*_m_) of BtGH84 in the presence of the initial activator **2** and several of the improved activators (Figure 3). Intriguingly, all the activators caused a decrease in the *T*_m_ value for BtGH84. This is in contrast to PUGNAc which increases *T*_m_ by 3 °C. This suggests that apo-BtGH84 is in fact destabilized by activator binding in the absence of any ligand in the active site. This may be a contributing factor to the difficulty of obtaining co-crystal structures of these compounds with BtGH84 in the absence of an active site ligand.[19]

**Figure 3:**
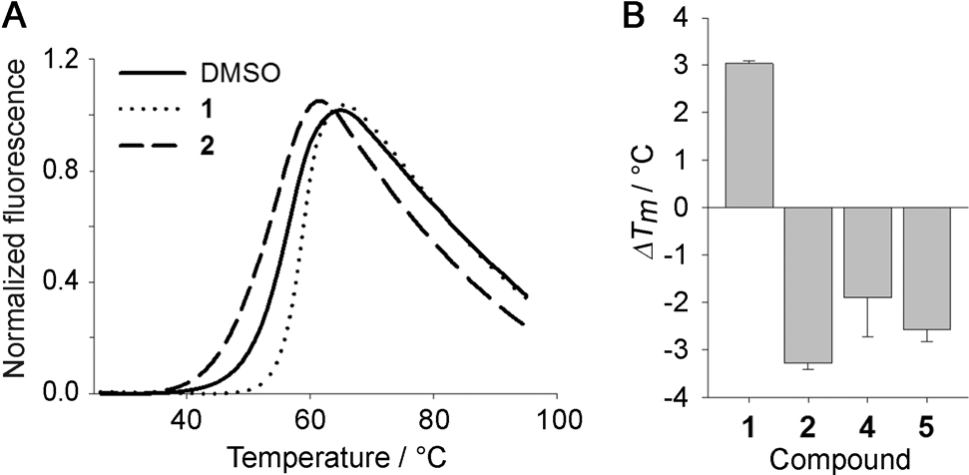
Activators have a destabilizing effect on apo-BtGH84. A) DSF curves for BtGH84 in the presence of inhibitor PUGNAc (1; 200 μm) and the initial activator 2 (8 mm), compared to BtGH84 in the presence of DMSO. B) Maximum shift in melting temperature from reference (Δ*T*_m_) for selected activators and PUGNAc (1).

The kinetics of BtGH84 activation are consistent with a nonessential type activation model (Scheme 1),[20] similar to reversible mixed inhibition. In this model, the dissociation constant of the substrate is modified in the presence of the activator (and vice versa); this modifier is denoted by the constant *α*. Additionally, the *k*_cat_ rate constant of the enzyme in the presence of the activator is modified by the constant *β*. These two values, *α* and *β*, allow the Michaelis–Menten equation to be adapted for a nonessential activator model (see Supporting Information). A series of kinetics experiments were performed with covariation of substrate and activator concentrations and the resulting data were fitted to this model. For an activator, *β* values greater than 1 show an increased catalytic rate in the presence of an activator and *α* values less than 1 show a decreased dissociation constant for a substrate in the presence of the activator. Together this leads to an enhanced rate of catalysis via the enzyme–substrate–activator complex. Covariation analysis for the thienopyrimidine activators were consistent with experiments (see above) at single activator concentrations. The change in substrate binding, indicated by the value of *α*, was 0.14 for activator **4** reflecting an approximate 7-fold enhancement of substrate binding in the presence of the activator (Figure S7 and Table S2). The *αK*_A_ values, which indicate the affinity of activator to the substrate-bound enzyme, were around 200 μm for the most effective activators **4** and **5**. Effects on the catalytic rate constant varied but the maximum effect was seen for **4** with *β*=1.7 which equates to a 1.7-fold increase of *k*_cat_ in the presence of the activator.

**Scheme 1:**
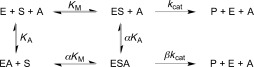
Nonessential reversible activator kinetic model. E=enzyme; S=substrate; A=activator; P=product.

Analysis of the crystal structure of BtGH84 allows speculation on the mechanism of activation. BtGH84 and other bacterial GH84 enzymes crystallize in either an “open” or “closed” form depending on the interactions made by any ligands. The loops that present the catalytic and substrate binding residues move between two distinct conformational states (Figure 2). In the closed form of BtGH84, Tyr137 (Y137) can form a hydrogen bond to stabilize the catalytic Asp243 (D243). This interaction is potentially enhanced by the π-stacking interaction with activator **2** seen in the crystal structure. Binding of the inhibitor PUGNAc also requires the closed form of BtGH84 (Figure S5) and through stabilizing this form activator **2** could increase the affinity for PUGNAc, as seen in the ITC and enzymatic assays described above. It is possible that the activators affect the enzyme–substrate complex in an analogous manner. Additionally, the destabilization of the apo enzyme by the activator compounds seen in the DSF experiments shows the activators promote a less stable conformation of the enzyme in the absence of other ligands. This could be either a closed conformation without the active site occupied, or an increased rate of interchange between the two forms. The structure and evidence of cooperative binding with PUGNAc is consistent with the kinetic data obtained from the activators that indicates they promote an enzyme–substrate complex of the enzyme thereby resulting in an increased rate of catalysis.

The activators showed no activation of a commercially available β-*N*-acetylglucosaminidase (Figure S8) demonstrating that the effect is specific. Additionally, there was no evidence of activation of human OGA (Figure S9); the sequence alignment between human OGA and BtGH84 (Figure S10) suggests that the equivalent residue to Arg347 in BtGH84 is Gln288 in human OGA. This alteration to a key point of interaction with the activators, as evident in the crystal structure, provides a structural rationale for the measured selectivity.

To our knowledge, this study has identified the first small molecules that directly activate a glycoside hydrolase enzyme. This activator was discovered directly from a biophysical fragment-based screening approach. Subsequent rational exploitation of commercially available analogues allowed the identification of activators that are effective at micromolar concentrations and which have an impact on the maximum rate of BtGH84, which the original hit did not. The effect of these activators on the enzyme kinetics and destabilization of the apo enzyme suggest that, in the case of BtGH84, this occurs through favoring the “closed” form of the active site which is the catalytically competent form of the enzyme.

With the majority of discovery efforts focused on enzyme inhibition, small-molecule enzyme activators remain an underexplored area of chemical probe development. However, the ability to tune the activity of an enzyme up, as well as down, has benefits for understanding the biological roles of a target and obtaining pharmacological insights. Activating an enzyme through the use of a small molecule has no clear equivalent in terms of gene manipulation as exogenous overexpression of a target of interest will not ensure a commensurate increase in enzyme activity.[21] In comparison to inhibition, the level of binding-site occupancy required for activators to have a pharmacologically relevant effect is extremely low. Enzyme inhibition is likely to require in-cell concentrations equivalent to 90–95 % inhibition whereas an activator concentration giving 10 % of maximum possible activity could be sufficient for a phenotypic effect.[22] Additionally, given the extensive use of glycoside hydrolases in industrial biotechnology, the observation that enzyme activators can be discovered should give strong impetus for their screening, discovery, and application across a large variety of industrial and medical sectors.

## Experimental Section

Gene expression of BtGH84, protein purification, and crystallization are essentially as described previously,[23] with further details in the Supporting Information.

Full methods and relevant references for NMR screening and further NMR experiments, ITC, 4MU-GlcNAc and *p*NP-GlcNAc enzymatic assays, DSF experiments, and structural determination can be found in the Supporting Information. The five activator compounds **2**–**6** were purchased from Enamine or Specs and confirmation of their identity and purity by LC-MS and ^1^H NMR spectroscopy can be found in the Supporting Information.
