# Supplementary material for: Discovery of Selective Small-Molecule Activators of a Bacterial Glycoside Hydrolase
Source: Angew Chem Int Ed Engl. 2014 Oct 7;53(49):13419–23. doi: 10.1002/anie.201407081 (PMC4501319; doi:10.1002/anie.201407081)
Supplement: Supplementary file 1 [file anie0053-13419-sd1.pdf]

Angewandte Chemie
Eine Zeitschrift der Gesellschaft Deutscher Chemiker

Supporting Information

© Wiley-VCH 2014

69451 Weinheim, Germany

Discovery of Selective Small-Molecule Activators of a Bacterial Glycoside Hydrolase**

*John F. Darby, Jens Landström, Christian Roth, Yuan He, Gideon J. Davies, and Roderick E. Hubbard**

anie_201407081_sm_miscellaneous_information.pdf

Table of Contents

Supplementary methods **2-5**
Supplementary figures **6-15**
Supplementary tables **16-17**
Compound characterisation **18-23**

Materials and Methods

Chemicals and reagents

Compounds in the fragment library were purchased from Specs, additional structural neighbours were purchased from Specs and Enamine. *O*-(2-Acetamido-2-deoxy-D-glucopyranosylidene) amino-*Z*-*N*-phenylcarbamate (PUGNAc) was purchased from Tocris Bioscience.

The fragment library was assembled as described by Schulz et al.[1] and dissolved in DMSO-d_6 to a concentration of 200 mM and quality controlled using ^{1}H NMR spectroscopy. Instant JChem 5.3.8, 2010, ChemAxon (http://www.chemaxon.com), was used for structure database management.

NMR spectroscopy and fragment screening

NMR experiments were carried out on a Bruker Avance II 700 MHz NMR spectrometer equipped with a triple resonance probe and z axis gradients. Data were processed using Topspin 3.2 software. Three NMR experiments were used for the screening, STD NMR,[2] PO-WaterLOGSY,[3] and $T_{1\rho}$-filtered[4] ^{1}H NMR experiments. All NMR screening data were collected at 288K and the samples contained 20 µM BtGH84, fragments at a concentration of approximately 200 µM per compound and 20 µM of TSP in 25 mM phosphate buffer containing 150 mM NaCl and 10% D_2O at pH 7.4. A single point titration by addition of PUGNAc was performed, giving a final PUGNAc concentration of 83 µM. The experiments were recorded using the standard STD NMR pulse sequence with a 15 ms 6 kHz spin-lock pulse to reduce the background protein resonances and excitation sculpting to suppress the water resonance. Saturation of the protein NMR signals of the enzyme were performed using a train of 70 selective 69 Hz Gaussian pulses with duration of 50 ms, adding up to a total saturation time of 3.5 s. The on-resonance frequency was set to 0.4 ppm and off-resonance irradiation was applied at 80 ppm. STD NMR spectra were acquired with a total of 480 transients in addition to 16 scans to allow the sample to come to equilibrium. Spectra were performed with a sweep width of 16 ppm and 16k data points corresponding to an acquisition time of 0.7 s and with an additional relaxation delay of 0.1 s giving a total repetition time of 4.3 s for the experiment. The PO-WaterLOGSY experiments were set-up using the same pulse sequence and parameters as Gossert *et al.*,[3] but with 128 transients, 32 'dummy' scans and a sweep width of 15 ppm. $T_{1\rho}$ filtered experiments were run with 32 transients, 16 'dummy' scans, a spin-lock pulse of 600 ms at 6 kHz and excitation sculpting to suppress the water resonance.

The affinity of the activator **2** was measured in the presence and absence of 200 µM PUGNAc, using a constant concentration of BtGH84 (8 µM), seven points of variable concentration of activator (0.07 – 12.5 mM), the same buffer as for the screening and at 298 K. The transverse relaxation rates, R_2, for the activator were measured at the different concentrations, using a CPMG pulse sequence with excitation sculpting to suppress the water resonance, 12 different spin-lock durations, 8-64 transients and a sweep width of 16 ppm. R_2 values and K_ds were then extracted using Sigmaplot.

Enzyme activity assay

AC_{50} measurements of fragment activators were performed using a fluorescent kinetic assay. Assays were carried out in a 96 well plate (Black Nunc F96 MicroWell Plates) at 25°C in a total volume of 100 µl per well, buffer (50 mM MES pH 6.5, 200 mM NaCl, 0.01% Tween-20, 1% DMSO) with 500 µM 4-methylumbelliferyl-β-D-GlcNAc (4MU-GlcNAc), as substrate and 50 nM BtGH84. Reactions were initiated by addition of 10 µl of substrate, to a final concentration of 500 µM, followed by 5 s of shaking. Fluorescent 4-methylumbelliferyl release was recorded continuously at 355 nm excitation and 460 nm emission for 20 s using a BMG PolarStar Optima plate reader. Rates were calculated using Sigmaplot as the slope of the curve of the collected data. Corrections were made to the detected gradients to adjust for the inner filter effect of 4MU-GlcNAc and convert the gradients into units of µmol min^{-1} according to a standard curve of 4MU. The initial slopes were fitted to four-parameter sigmoidal dose response curves using SigmaPlot and the AC_{50} values extracted. Inhibition of BtGH84 by PUGNAc (IC_{50}) was determined in similar manner to the AC_{50} values except that enzyme activity was normalised in the absence and presence of the activator **2**.The absorbance assay was performed under similar conditions with 500 µM *p*NP-GlcNAc (4-nitrophenyl-β-D-GlcNAc) as substrate and *p*NP release recorded by absorbance at 400 nm.

Kinetic experiments using 4MU-GlcNAc were carried out in an analogous manner except that reactions were initiated by a 10 µl injection of BtGH84, to a final concentration of 50 nM, in a well containing the substrate at the desired concentration. Values quoted throughout are mean and standard deviation of three independent replicates.

Apparent K_M and V_{max} values were calculated from a Michalis-Menten curve fit to 8-10 point substrate titrations at a constant concentration of the activator. The covariation experiments used the same range of substrate concentrations across an activator concentration range of 50 µM to 2 mM. The data obtained from these experiments was fit to the nonessential activator equation:

$$v = \frac{V_{max}[S]}{K_M\left[\dfrac{1+\dfrac{[A]}{K_A}}{1+\dfrac{\beta[A]}{\alpha K_A}}\right] + [S]\left[\dfrac{1+\dfrac{[A]}{\alpha K_A}}{1+\dfrac{\beta[A]}{\alpha K_A}}\right]}$$

Here, [S] is the concentration of the enzyme, [A] the concentration of the activator, α modifies K_M, β modifies V_{max} and K_A is the half-max concentration of the activator.

ITC

Isothermal titration calorimetry (ITC) measurements were carried out using a VC calorimeter (Microcal, Northampton, MA) at 25°C. BtGH84 was dialysed against 50 mM MES, pH 6.5, 200 mM NaCl, and concentrated to 70 and 100 µM for experiments with 10 mM and 0 mM of **2** respectively. Samples were centrifuged and degassed prior to use. PUGNAc was diluted in the same buffer to a final concentration of 0.8 respectively 1.2 mM and degassed. Titrations were performed by injection of 10 µL aliquots of the inhibitor into the BtGH84 solution. Using Microcal Origin software, the enthalpy for each injection was plotted against the molar ratio and fitted to a bimolecular model. The stoichiometry (n), enthalpy (ΔH), and association

constant (K_a) were obtained from this fitting, and were used to calculate the Gibbs free energy (ΔG) and entropy (TΔS).

Crystallisation and structure determination

BtGH84 crystals were grown using the sitting-drop vapour diffusion method. 1 µl of 12 mg/ml BtGH84 was mixed with 1 µl of reservoir solution (0.1 M imidazole pH 8.0, 10% PEG8000 (w/v), 3% trimethylamine N-oxide dehydrate, 12% ethylene glycol) and the crystals were allowed to grow at room temperature for at least 2 days. Crystal soaking was performed by gradually adding well solution containing 10 mM PUGNac and 50 mM activator **2**, and 10% DMSO. Crystals were picked up with a nylon fibre loop (Hampton Research) and plunged into liquid nitrogen. Data were collected at Diamond beamline I04 using a Pilatus 6M-F detector (Dectris) at 100 K. An oscillation range of 0.1° degree was chosen and 180° were collected. Data were indexed, integrated and scaled using XDS.[5] Indexing was chosen to be consistent with previous collected data using Pointless[6] and a direct refinement of the model against the data was carried out. The model was improved and adjusted by alternating cycles of manual rebuilding and real-space refinement in COOT[7] and reciprocal-space refinement in Refmac[8] and Phenix.[9] Coordinate files and libraries for the ligand were created using phenix.eLBOW.[9] The quality of the final model was evaluated using the MolProbity-Server.[10] Figures were created using PyMol (Schrodinger LLC).

Differential scanning fluorimetry

DSF experiments were performed in 96-well polypropylene PCR plates (Agilent 401334) with a final well volume of 25 µl. Well components were BtGH84 (2 µM), Sypro Orange dye (2X), DMSO (2%), and compound at either 2, 4 or 8 mM dependent on solubility in the same buffer as used for the enzymatic assays. Melting data was collected using the Agilent Stratagene Mx3005P rtPCR machine ramping from 25°C to 95°C at 30 sec per degree. Data was analysed with a combination of the Agilent MxPro software, MTSA,[11] and the DSF analysis spreadsheet in Excel.[12]

Human OGA expression

Human OGA was expressed and purified in an analogous manner to that previously described by Macauley et al.[13]

References

[1] M. N. Schulz, J. Landstrom, K. Bright, R. E. Hubbard, *J Comput Aided Mol Des* **2011**, *25*, 611-620.

[2] M. Mayer, B. Meyer, *Angewandte Chemie International Edition* **1999**, *38*, 1784-1788.

[3] A. D. Gossert, C. Henry, M. J. Blommers, W. Jahnke, C. Fernandez, *J Biomol NMR* **2009**, *43*, 211-217.

[4] P. J. Hajduk, E. T. Olejniczak, S. W. Fesik, *Journal of the American Chemical Society* **1997**, *119*, 12257-12261.

[5] W. Kabsch, Acta crystallographica. Section D, Biological crystallography **2010**, 66, 125-132.

[6] P. R. Evans, *Acta crystallographica. Section D, Biological crystallography* **2011**, *67*, 282-292.

[7] P. Emsley, B. Lohkamp, W. G. Scott, K. Cowtan, *Acta crystallographica. Section D, Biological crystallography* **2010**, *66*, 486-501.

[8] G. N. Murshudov, A. A. Vagin, E. J. Dodson, *Acta crystallographica. Section D, Biological crystallography* **1997**, *53*, 240-255.

[9] P. D. Adams, P. V. Afonine, G. Bunkoczi, V. B. Chen, I. W. Davis, N. Echols, J. J. Headd, L. W. Hung, G. J. Kapral, R. W. Grosse-Kunstleve, A. J. McCoy, N. W. Moriarty, R. Oeffner, R. J. Read, D. C. Richardson, J. S. Richardson, T. C. Terwilliger, P. H. Zwart, *Acta crystallographica. Section D, Biological crystallography* **2010**, *66*, 213-221.

[10] V. B. Chen, W. B. Arendall, 3rd, J. J. Headd, D. A. Keedy, R. M. Immormino, G. J. Kapral, L. W. Murray, J. S. Richardson, D. C. Richardson, *Acta crystallographica. Section D, Biological crystallography* **2010**, *66*, 12-21.

[11] M. N. Schulz, J. Landstrom, R. E. Hubbard, *Analytical biochemistry* **2013**, *433*, 43-47.

[12] F. H. Niesen, H. Berglund, M. Vedadi, *Nature protocols* **2007**, *2*, 2212-2221.

[13] M. S. Macauley, K. A. Stubbs, D. J. Vocadlo, *J Am Chem Soc* **2005**, *127*, 17202-17203.

Supplementary figures

Figure S1: Chemical structures of hits identified from NMR-based fragment screening of BtGH84. The hits are divided into those competitive with the inhibitor PUGNAc (upper box) and those which were not competitive (lower box).

Competitive with PUGNAc (IC$_{50}$ values in brackets where obtained)

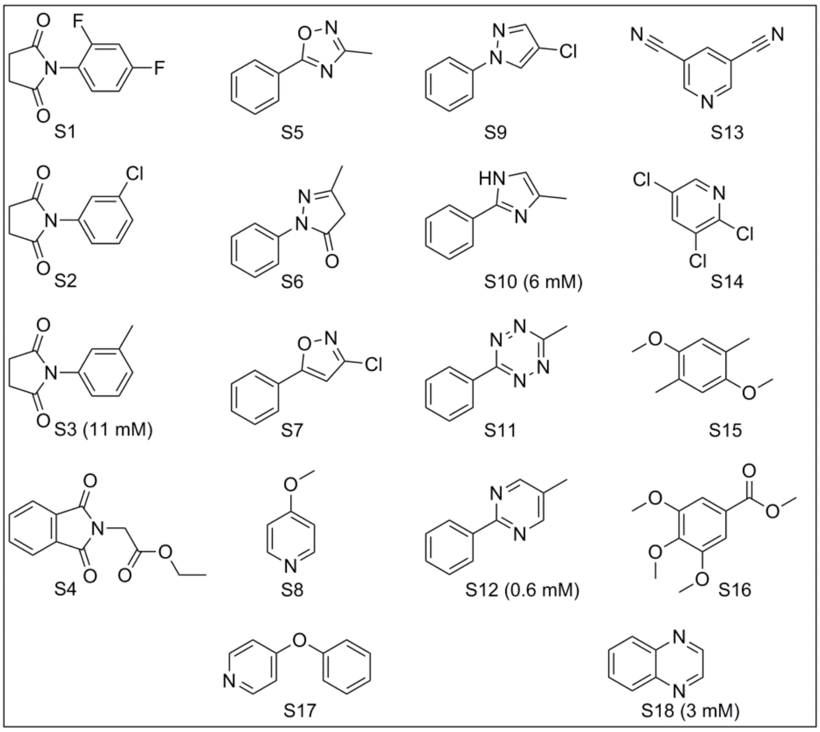

Non-competitive with PUGNAc

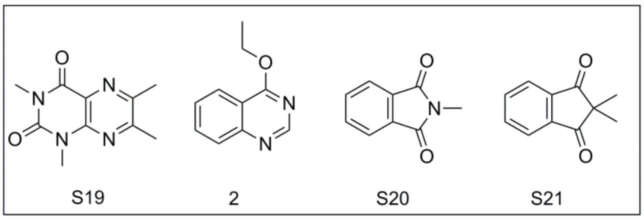

Figure S2. STD NMR spectrum of **2** (800 µM) with BtGH84 (8 µM) shown in black and following addition of PUGNAc (108 µM) shown in red. (A) Shows the entire spectrum and (B) shows 7.6 – 8.8 ppm where several of the peaks appear to increase in intensity following the addition of PUGNAc.

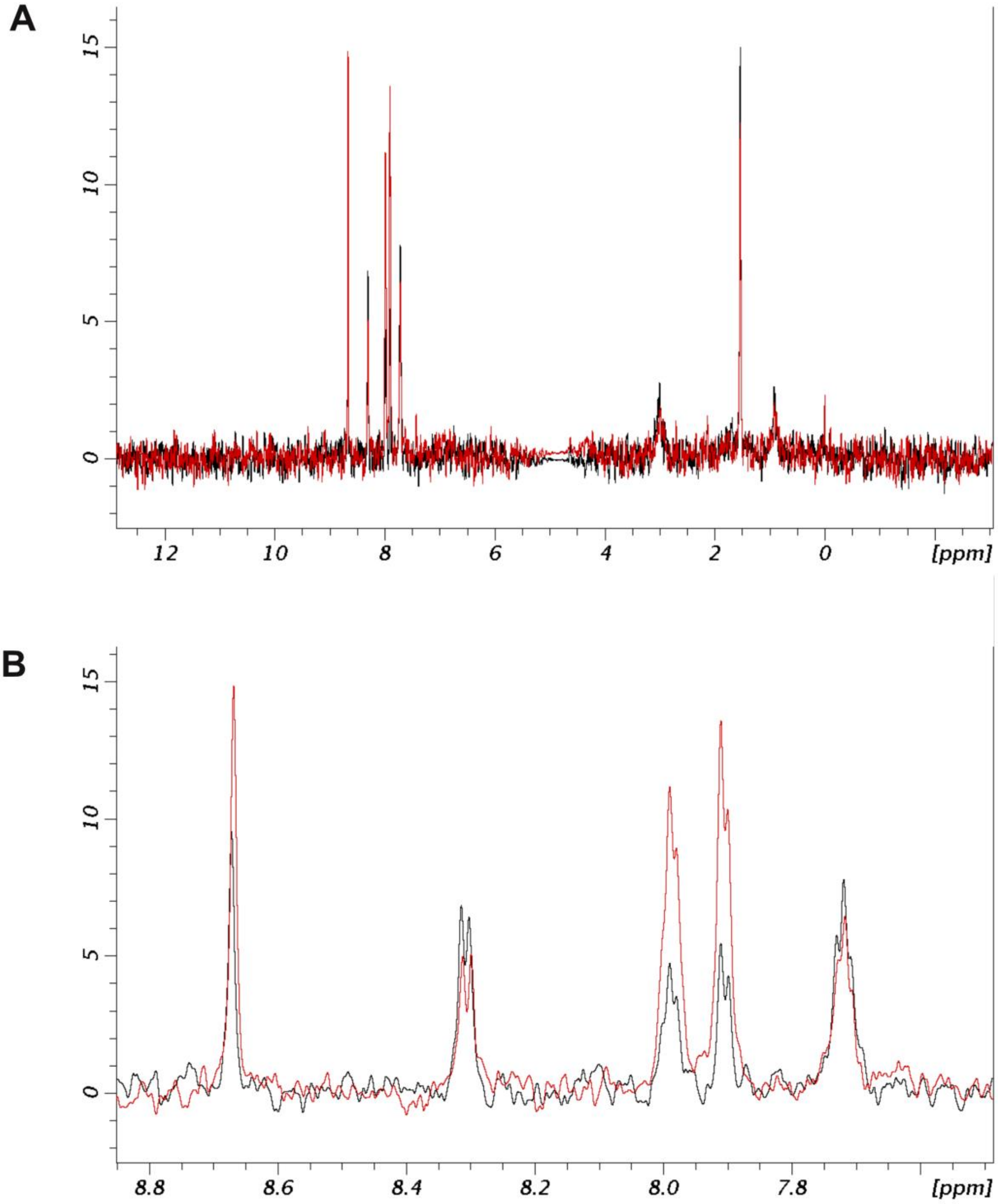

Figure S3. Dissociation constant of **2** for BtGH84 determined in the presence of PUGNAc with ^{1}H R_2 relaxation rates. The R_2 relaxation rate is plotted versus activator **2** concentration in the presence of 8 µM BtGH84 and 200 µM PUGNAc. Data is fitted with a four parameter logistic in Sigmaplot and the inflection point is taken as the K_d.

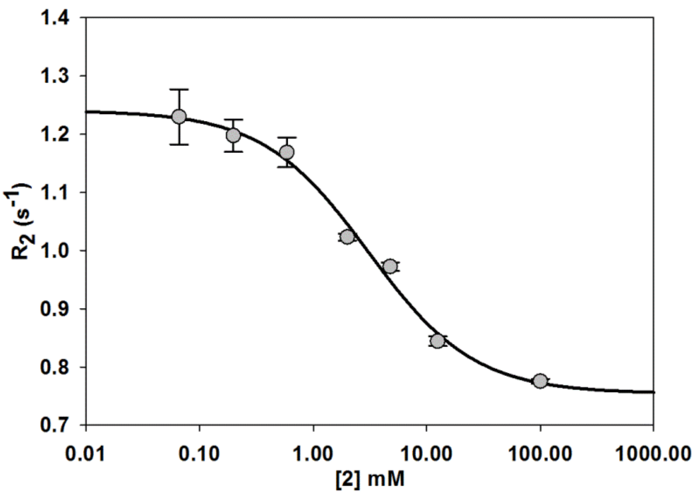

Figure S4. (A and B): ITC data for PUGNAc binding to BtGH84 in the absence (A) and presence (B) of the activator **2**. (C and D) IC_{50} curve of BtGH84 inhibition by PUGNAc (**1**) in 4MU-GlcNAc cleavage assay. PUGNAc IC_{50} is reduced in the presence of 4mM activator **2.**

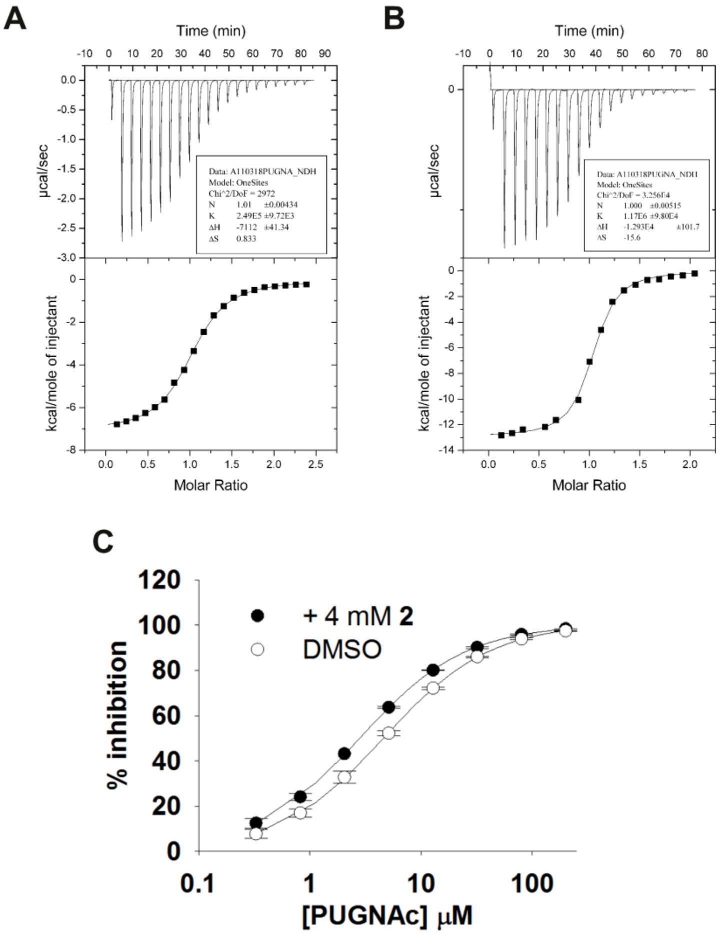

D

	**2**	DMSO
IC_{50} (µM)	2.8 ± 0.22	4.3 ± 0.19
Hillslope	0.92	0.90

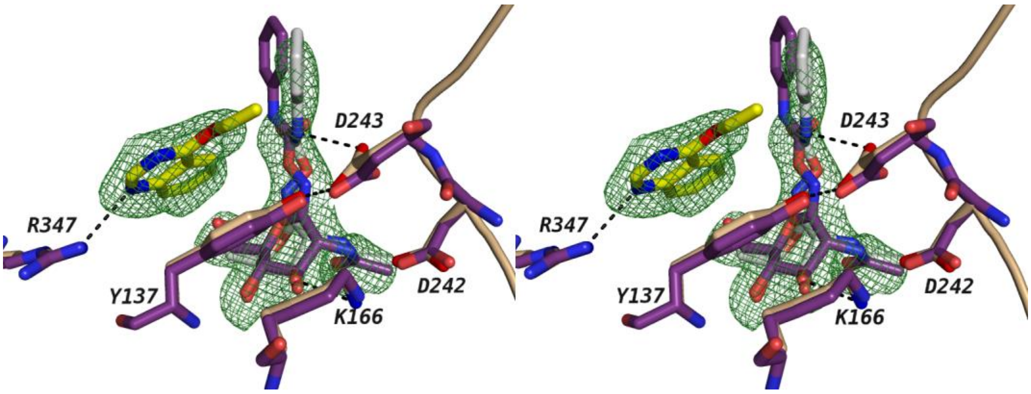

Figure S5. Stereo image of the BtGH84 active site with PUGNAc bound (purple, PDB:2VVS) overlaid with BtGH84 bound to activator **2** and PUGNAc (coloured as in Figure 2, PDB:4UR9) with the SA-Fo-Fc omit map contoured at 3σ r.m.s. With PUGNAc alone the amide linking the phenyl ring is rotated 180° from the structure with the activator, this results in the loss of a hydrogen bond from D243 seen in the activator structure.

Figure S6. STD NMR spectrum of activator **5** (250 μM) in the presence of BtGH84 (10 μM) is shown in black, the same experiment with 25 mM of **2** as a competitor is shown in red. There is a clear loss in signal intensity of **5** with an excess of **2** suggesting reduced binding to BtGH84.

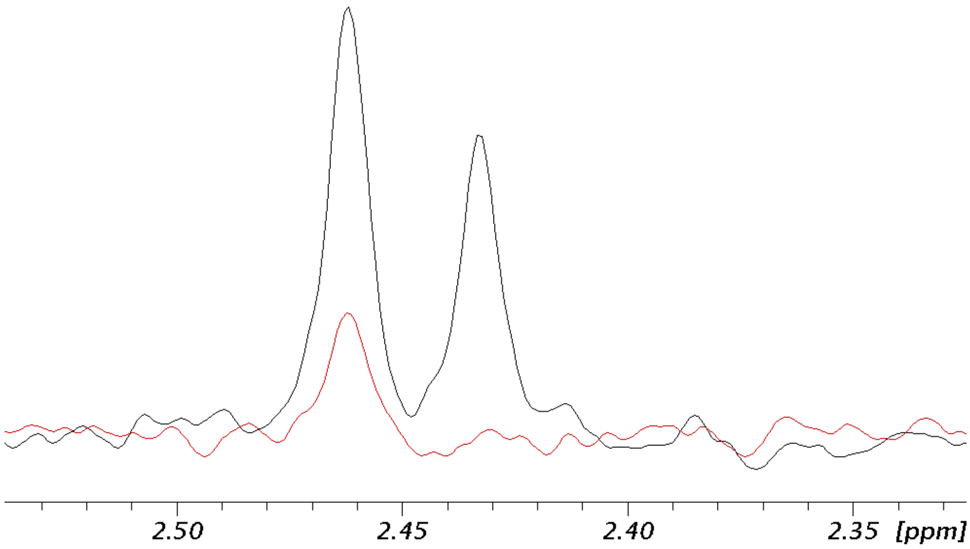

Figure S7: Covariation curve fits to the non-essential activator model for compounds **3**, **4** and **5**. See supplementary methods for analysis details. The highest concentration of activator **3** attainable at 1% DMSO (2 mM) does not appear to be approaching a saturating concentration. As a result the fitted values for α and K_a are not accurate for this data set.

Activator **3**.

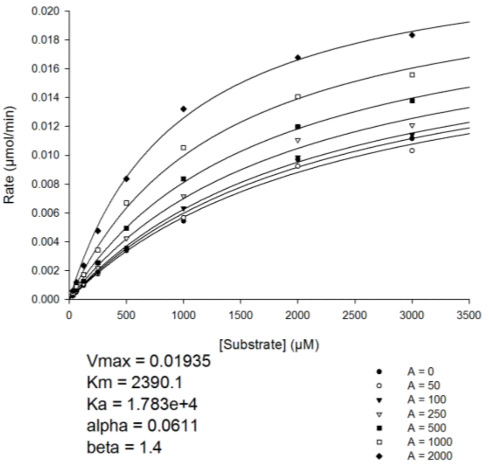

Activator **4**.

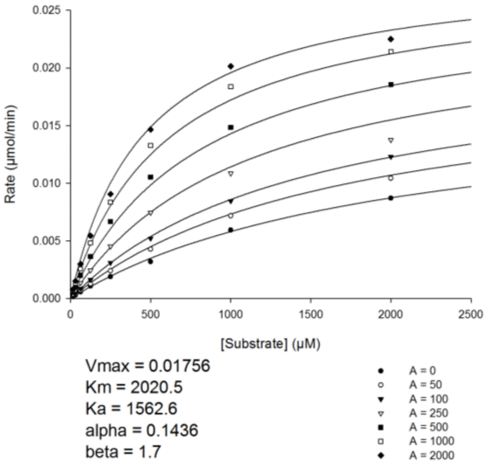

Activator **5**.

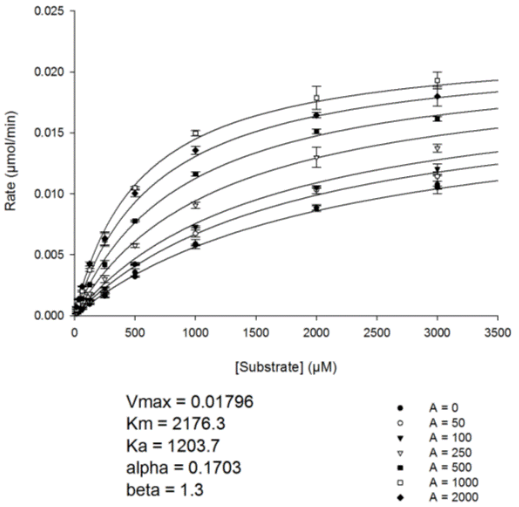

Figure S8: Effect of activators **2** and **4** on a β-N-Acetylglucosaminidase from *Canavalia ensiformis* (Jack bean). (A) Titration of the 4MU-GlcNAc substrate at the optimum pH suggested for this enzyme (pH 4.9) and (B) titration at the same pH as used for the BtGH84 studies (pH 6.5). This demonstrates that activators **2** and **4** do not increase the activity of this enzyme. Enzyme supplied by Sigma (purified from native source).

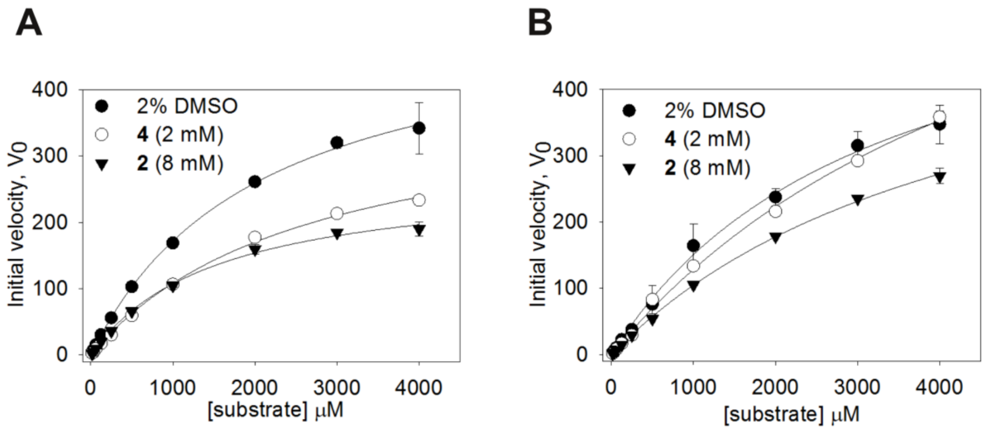

Figure S9: Effect of activators **2** and **4** on human OGA. Titration of the 4MU-GlcNAc substrate at pH 6.5. Activators **2** and **4** do not appear to increase the activity of this enzyme.

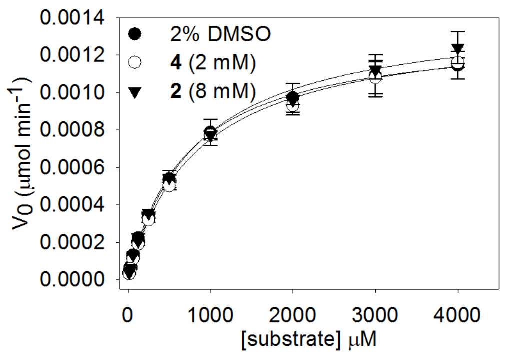

Figure S10: Sequence alignment between catalytic domain of human OGA and BtGH84. Sequence alignment generated for the catalytic domains of BtGH84 and hOGA using the T-Coffee service (http://tcoffee.crg.cat/apps/tcoffee/index.html) and the figure generated with ESPript 3.0 (http://espript.ibcp.fr/ESPript/ESPript/) where identical residues are highlighted in red, similar residues in yellow, and secondary structure as arrows for beta sheet and a spring for a helix. The residue numbering is that for BtGH84.

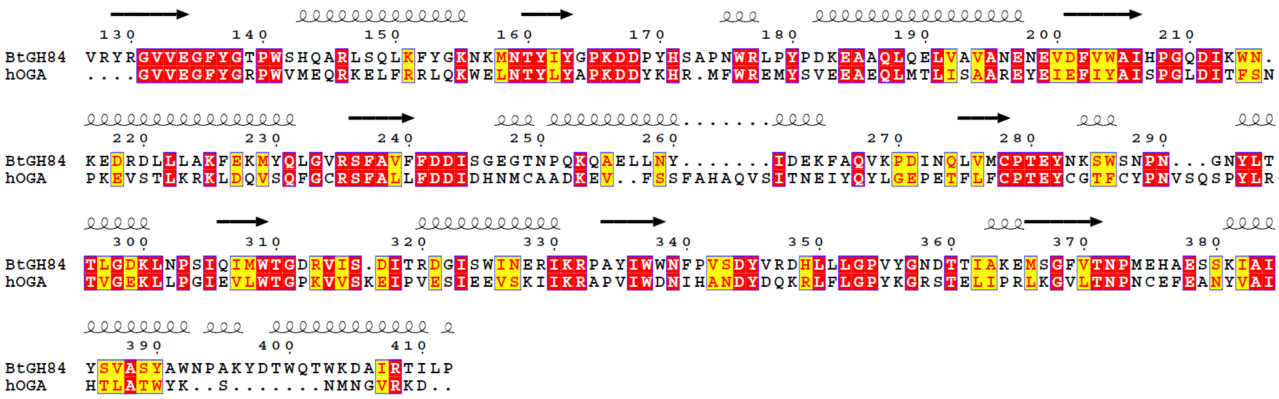

Supplementary tables

Table S1: Data collection and refinement statistics

	**BtGH84_activator**
X-ray-source	Diamond I04
wavelength [Å]	0.976250
space group	$P22_12_1$
Resolution limit	2.2
Unit cell parameters (a , b, c) [Å]	51.49, 162.74, 223.18
Solvent content	0.556
Monomers per au	2
Total reflections	644525
Unique reflections	183412
I/σ(I)	6.8.7(1.0)
completeness	0.997(0.969)
R_{merge}	0.030(2.224)
R_{rim}	0.034(2.414)
R_{pim}	0.015(0.930)
$CC^{1/2}$	0.997(0.526)
**Refinement**	
R_{cryst}	0.222
R_{free}	0.252
Number of	
Protein residues	1352
Ligand atoms	76
Water	405
Ions	1
r.m.s.d.	
Bond [Å]	0.004
Angle [°]	0.774
Average B-Factor [Å^2]	
Protein	62.8
Ligand	46.9
Water	51.6
Ramachandran plot [%]	
Favored/Allowed/Disallowed	96.5/3.4/0.1
PDB accession code	4UR9

Table S2. Selected parameters for activation of 4MU-GlcNAc cleavage by BtGH84 by thienopyrimidine activators fit using the non-essential activator equation to data from covariation experiments.

Compound	Vmax (μmol min^{-1})	K_M (μM)	K_A (μM)	α	β	αK_A (μM)	βk_{cat} (s^{-1})
**3**	0.019 ± 0.0007	2390 ± 163	17,830 ± 2,650	0.061 ± 0.04	1.4 ± 0.1	1089	88.7
**4**	0.018 ± 0.002	2020 ± 159	1563 ± 177	0.14 ± 0.02	1.7 ± 0.1	224	100
**5**	0.018 ± 0.0008	2176 ± 171	1204 ± 166	0.17 ± 0.02	1.3 ± 0.07	205	75.8

Characterisation of activator compounds 2-6

Compounds **2**-**5** were purchased from Specs and compound **6** from Enamine. The supplied compounds were prepared as 200 mM stocks in d_6-DMSO. Identify and purity of these compounds was confirmed by ^{1}H NMR and LCMS analysis. NMR data collected as detailed in the Supplementary experimental data, buffer and solvent peaks can be seen at 3.34 and 2.68 ppm for methanol and DMSO respectively. The intensity of peaks from 4-5 ppm may be lower than expected due to water suppression. LC/MS data were obtained on a Bruker micrOTOF mass spectrometer with electrospray capability coupled to an Agilent 1200 series LC system.

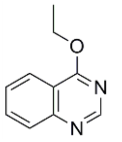

2 (4-ethoxyquinazoline). ^{1}H NMR (700 MHz, 80 mM sodium phosphate pH 6.7, 1.75% d_6-DMSO, D_2O, 25°C, DSS): δ=8.63 (s, 1H, C(2)H), 8.26 (d, ^{3}J(H,H)=8.8 Hz, 1H, C(5)H), 7.96 (dd, ^{3}J(H,H)=8.2, 8.0 Hz, 1H, C(7)H), 7.87 (d, ^{3}J(H,H)=8.3 Hz, 1H, C(8)H), 7.69 (dd, ^{3}J(H,H)=8.0, 8.1 Hz, 1H, C(6)H), 4.60 (q, ^{3}J(H,H)=7.1 Hz, 2H, CH_2), 1.52 (t, ^{3}J(H,H)=7.1 Hz, 3H, CH_3). LC/MS (ESI): R_t = 20, *m/z* $[M+H]^+$ calc: 175.0866 found: 175.0872.

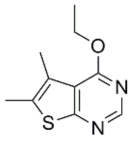

3 (4-ethoxy-5,6-dimethylthieno[2,3-d]pyrimidine). ^{1}H NMR (700 MHz, 80 mM sodium phosphate pH 6.7, 1.75% d_6-DMSO, D_2O, 25°C, DSS): δ=8.35 (s, 1H, C(2)H), 4.46 (q, ^{3}J(H,H)=7.2 Hz, 2H, CH_2), 2.43 (s, 3H, C(6)Me), 2.37 (s, 3H, C(5)Me), 1.46 (t, ^{3}J(H,H)=7.2 Hz, 3H, CH_3). LC/MS (ESI): R_t = 44, *m/z* $[M+H]^+$ calc: 209.0743 found: 209.0736

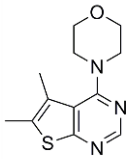

4 (4-{5,6-dimethylthieno[2,3-d]pyrimidin-4-yl}morpholine). ^{1}H NMR (700 MHz, 80 mM sodium phosphate pH 6.7, 1.75% d_6-DMSO, D_2O, 25°C, DSS): δ=8.36 (s, 1H, C(2)H), 3.93 (t, ^{3}J(H,H)=4.7 Hz, 4H, CH_2OCH_2), 3.42 (t, ^{3}J(H,H)=4.7 Hz, 4H, CH_2NCH_2), 2.45 (s, 3H, C(6)Me), 2.41 (s, 3H, C(5)Me). LC/MS (ESI): R_t = 34, *m/z* $[M+H]^+$ calc: 250.1009 found: 250.1003.

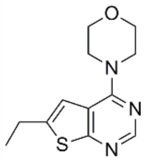

5 (4-{6-ethylthieno[2,3-d]pyrimidin-4-yl}morpholine). ^{1}H NMR (700 MHz, 80 mM sodium phosphate pH 6.7, 1.75% d_6-DMSO, D_2O, 25°C, DSS): δ=8.31 (s, 1H, C(2)H), 7.20 (s, 1H, C(5)H), 3.89 (t, ^{3}J(H,H)=4.7 Hz, 4H, CH_2OCH_2), 3.87 (t, ^{3}J(H,H)=4.7 Hz, 4H, CH_2NCH_2), 2.93 (q, ^{3}J(H,H)=7.8 Hz, 2H, C(6)CH_2), 1.32 (t, ^{3}J(H,H)=7.8 Hz, 3H, CH_3). LC/MS (ESI): R_t = 35, *m/z* $[M+H]^+$ calc: 250.1009 found: 250.1017.

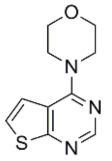

6 (4-{thieno[2,3-d]pyrimidin-4-yl}morpholine). ^{1}H NMR (700 MHz, 80 mM sodium phosphate pH 6.7, 1.75% d_6-DMSO, D_2O, 25°C, DSS): δ=8.38 (s, 1H, C(2)H), 7.56 (d, ^{3}J(H,H)=6.1 Hz, 1H, C(5)H), 7.53 (d, ^{3}J(H,H)=6.1 Hz, 1H, C(6)H), 3.94 (t, ^{3}J(H,H)=4.5 Hz, 4H, CH_2OCH_2), 3.90 (t, ^{3}J(H,H)=4.5 Hz, 4H, CH_2NCH_2). LC/MS (ESI): R_t = 15, *m/z* $[M+H]^+$ calc: 222.0696 found: 222.0696.

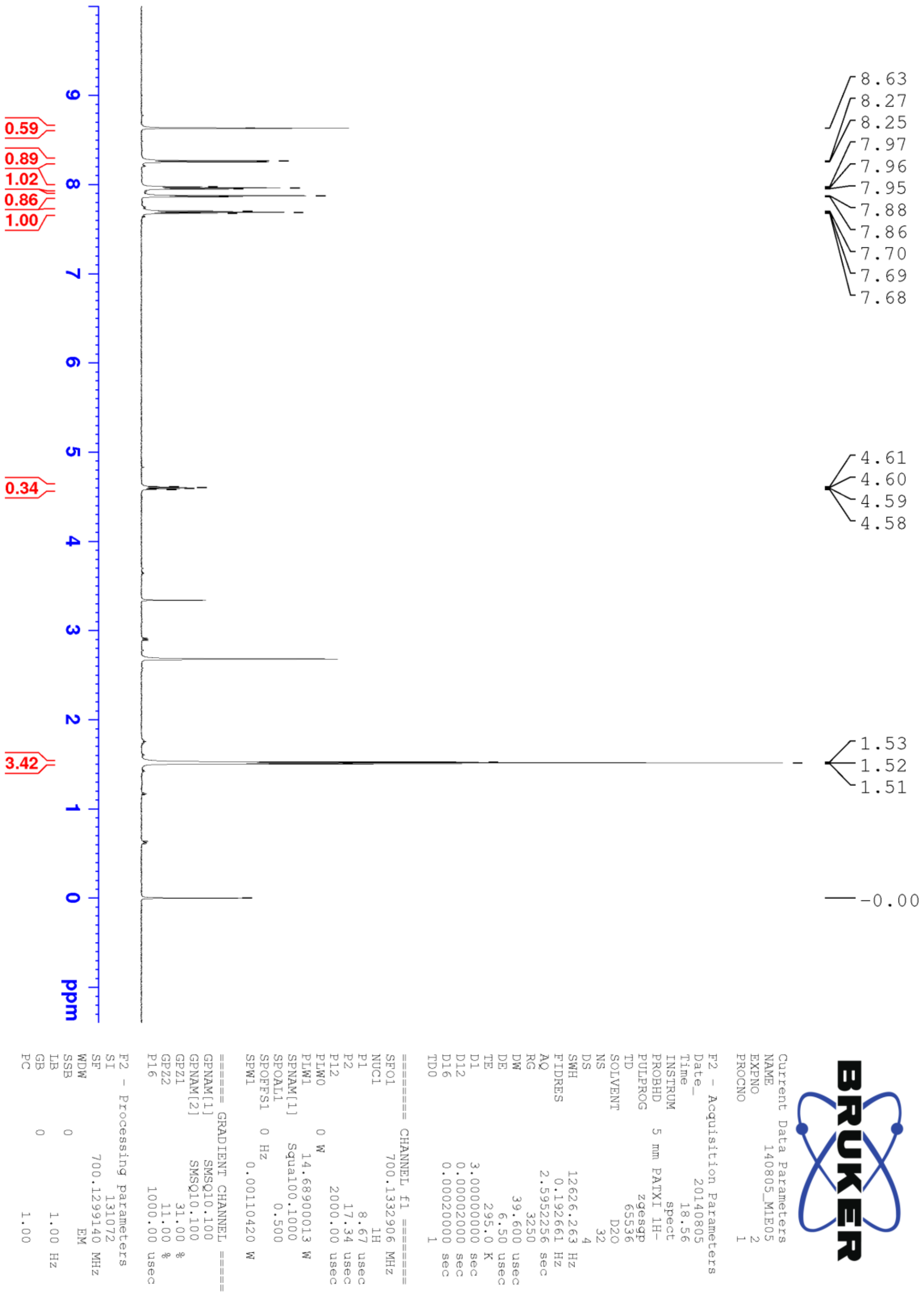

Compound **2** ^{1}H NMR spectrum.

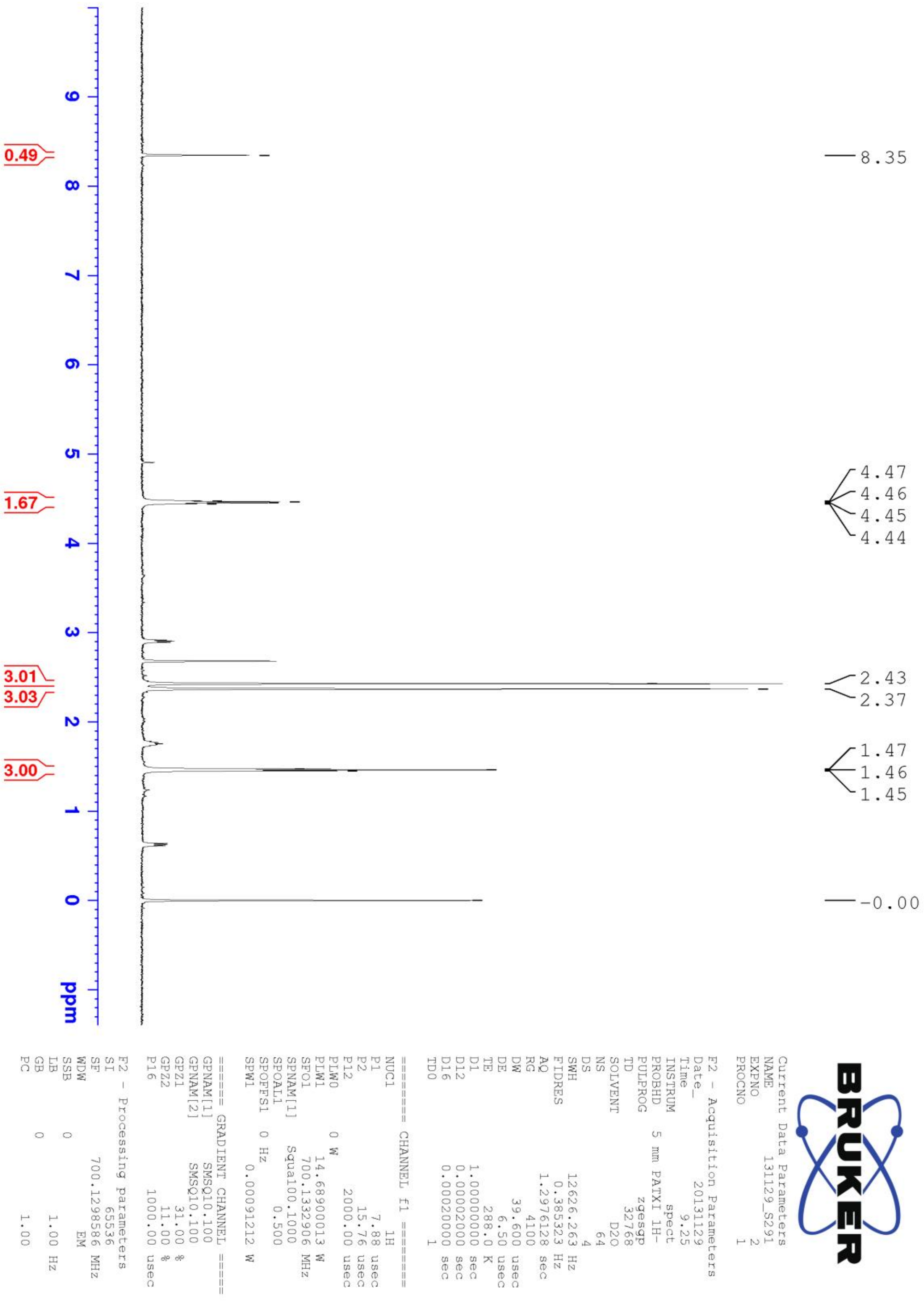

Compound **3** ^{1}H NMR spectrum.

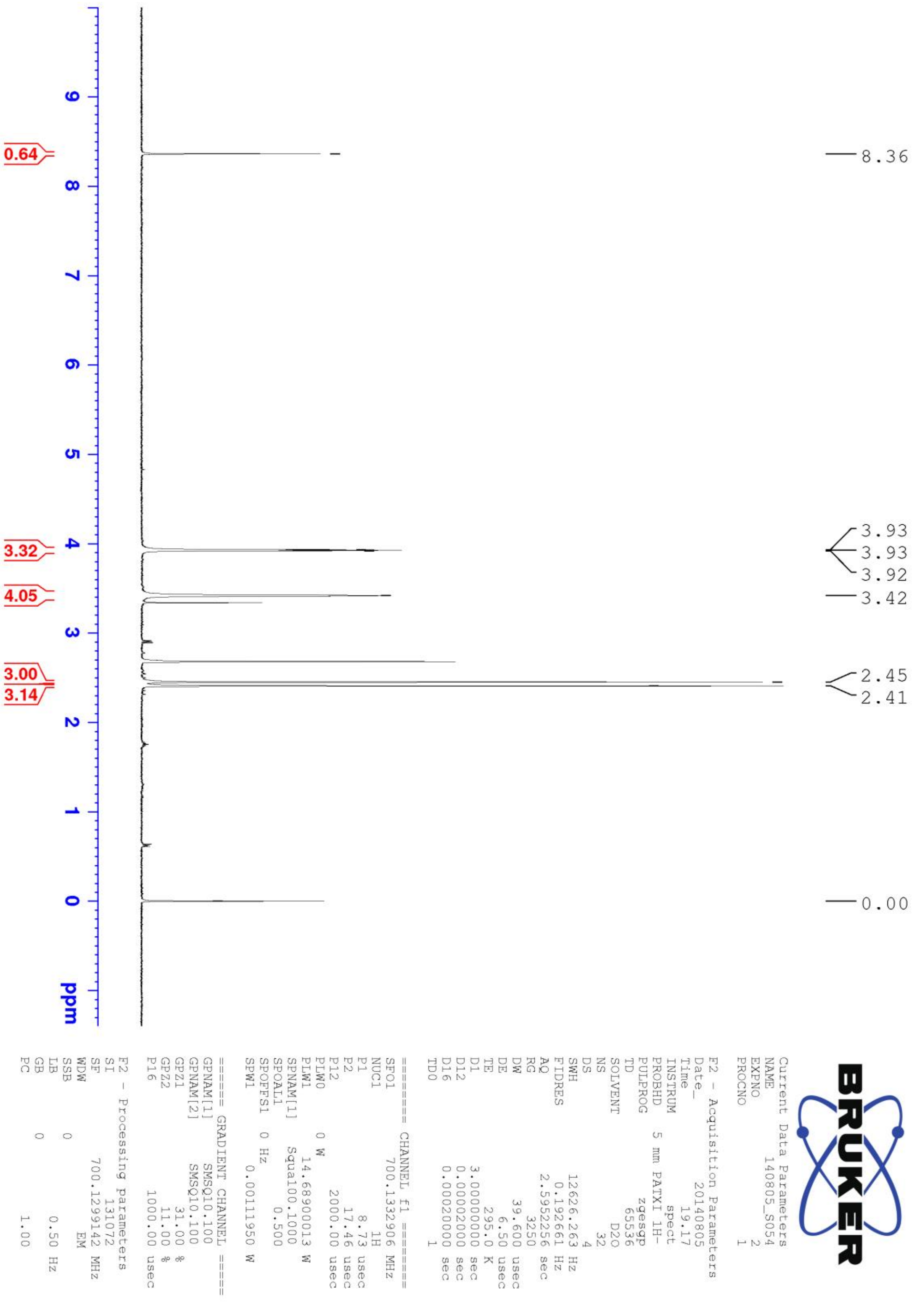

Compound **4** ^{1}H NMR spectrum.

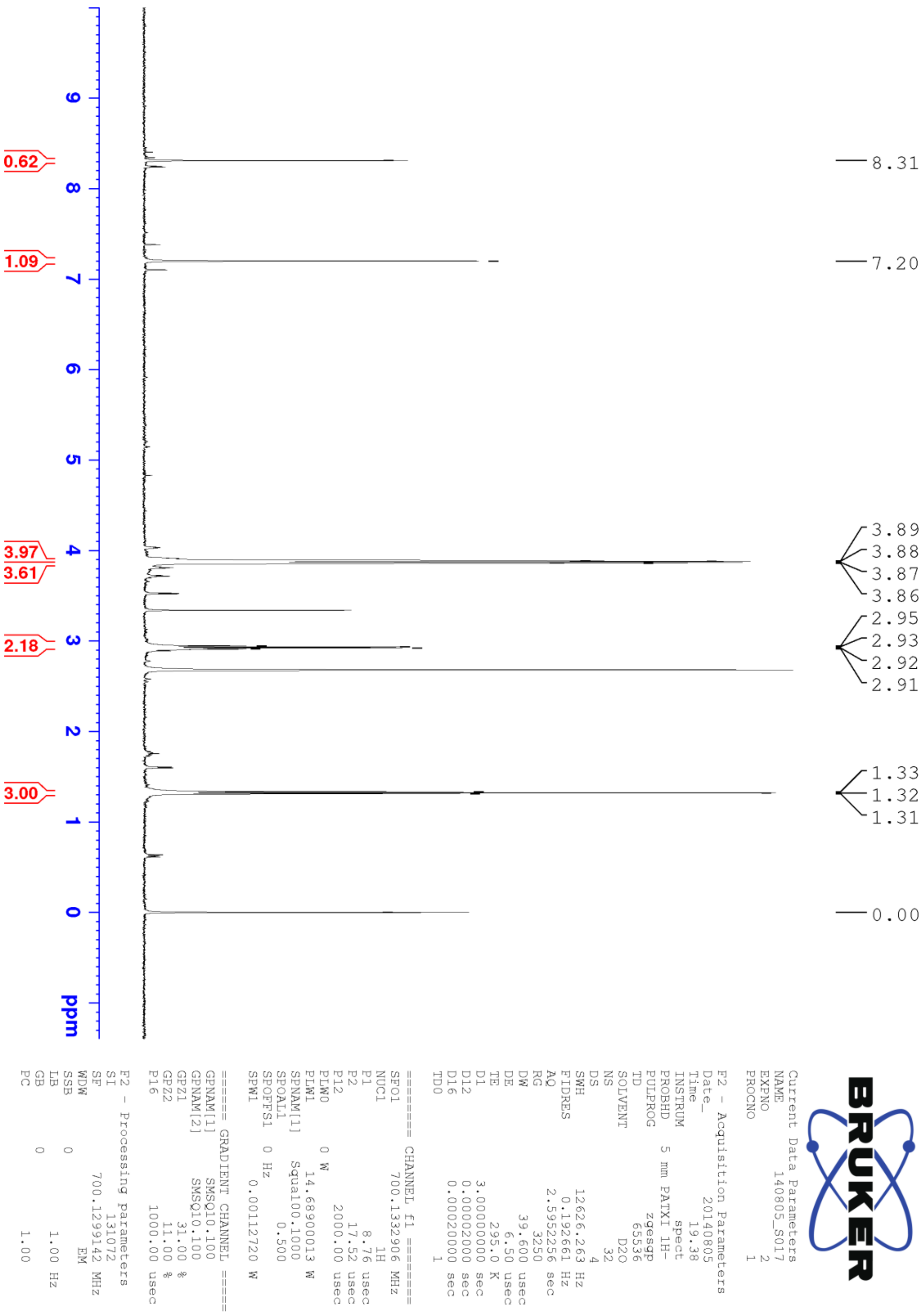

Compound **5** ^{1}H NMR spectrum.

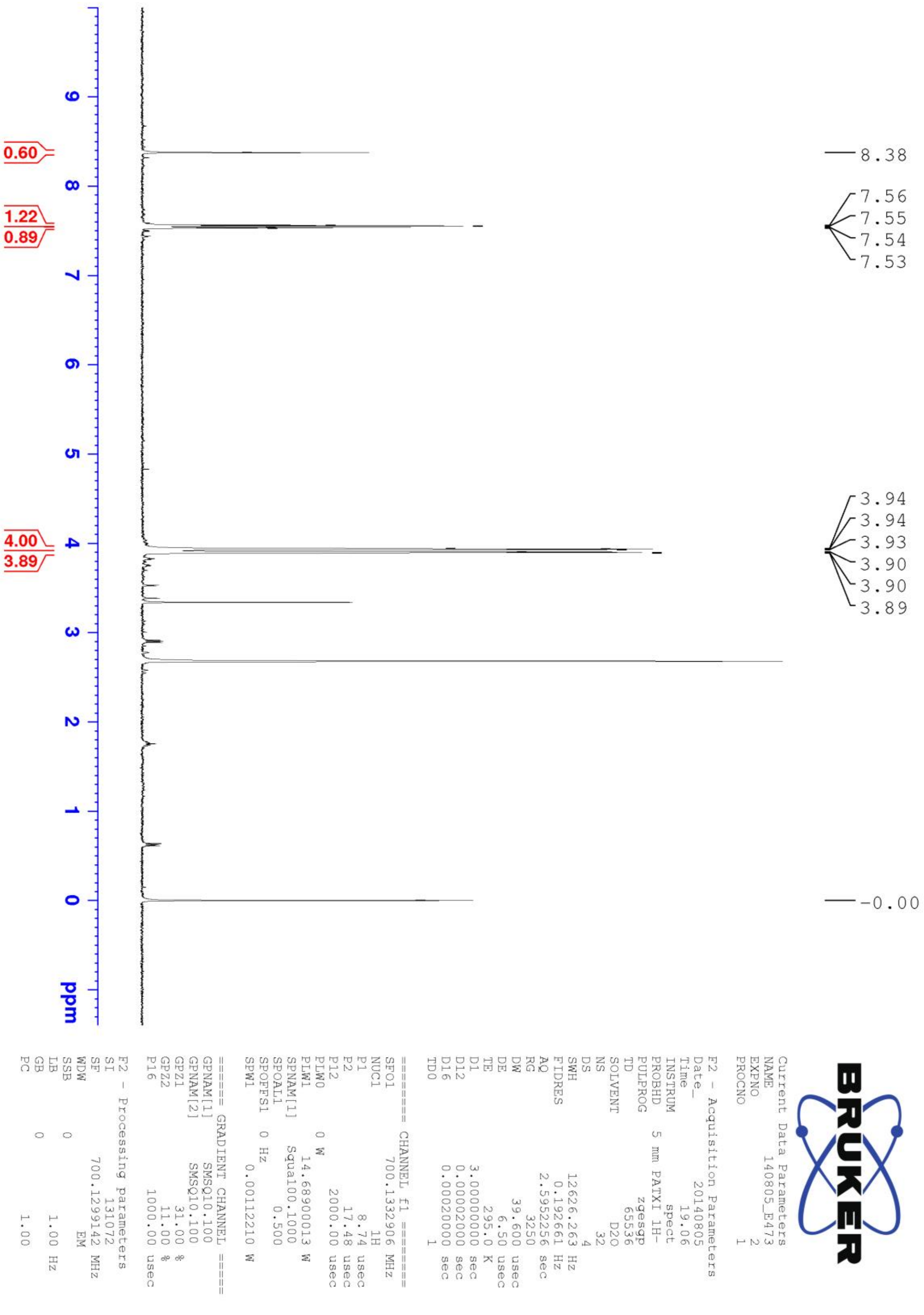

Compound **6** ^{1}H NMR spectrum.
